# Herbal formula alleviates heat stress by improving physiological and biochemical attributes and modulating the rumen microbiome in dairy cows

**DOI:** 10.3389/fvets.2025.1558856

**Published:** 2025-03-07

**Authors:** Xiaofang Wang, Yawen Wang, Man Feng, Jiefeng Li, Ze Liu, Le Fu, Ning Zhang, Huaying Zhang, Jianhua Qin

**Affiliations:** ^1^College of Veterinary Medicine, Hebei Agricultural University, Baoding, China; ^2^Institute of Animal Husbandry and Veterinary Medicine of Hebei, Baoding, China; ^3^Chengde Academy of Agriculture and Forestry Sciences, Chengde, China; ^4^Beijing-Tianjin-Hebei Modern Agriculture Collaborative Innovation and Development Service Center, Baoding, China

**Keywords:** heat stress, dairy cow, herbal formula, antioxidant capacity, immune response, rumen microbiome

## Abstract

Heat stress significantly impacts dairy cow productivity, health, and welfare. This study evaluated a self-developed herbal formula as a dietary intervention to mitigate heat stress. A total of 198 lactating cows were divided into two groups: a Control group receiving standard total mixed rations and a Herbs group supplemented with herbal formula for 60 days. Various parameters were assessed, including milk yield and composition, antioxidant capacity, immune responses, stress-related gene expression, and rumen microbial composition. Compared to the Control group, cows in the Herbs group showed improved feed intake, milk yield and quality, rumination frequency, and enhanced antioxidant activity and immune response. Rumen microbiome analysis revealed a reduced relative abundance of Proteobacteria and *Ochrobactrum* in the Herbs group, along with an enrichment of beneficial genera such as *Lachnospira*. Functional predictions indicated that the Herbs group exhibited enhanced glycolysis/gluconeogenesis, pyruvate metabolism, and starch and sucrose metabolism, reflecting improved fermentation efficiency and energy utilization. In conclusion, the herbal formula improved physiological and biochemical attributes, boosted antioxidant and immune responses, and modulated the rumen microbiome, contributing to the alleviation of heat stress in dairy cows. These findings highlight its potential as a natural dietary strategy to support dairy cow health and productivity under heat stress conditions.

## 1 Introduction

Heat stress is a critical challenge faced by the dairy industry worldwide, significantly compromising milk production, reproductive efficiency, and overall animal health (1–3). Dairy cows are particularly vulnerable to rising environmental temperatures due to their intensive metabolic heat production and small surface:volume ratio (4). Heat stress not only disrupts the physiological and hormonal balance of cows but also alters their feeding behavior, leading to reduced feed intake and nutrient absorption (2, 5, 6). In recent years, dietary strategies have emerged as an effective approach to mitigate the negative impacts of various types of stress (7–9). Among these, herbal supplements have gained increasing attention due to their potential to enhance antioxidant capacity, modulate immune responses, and regulate stress-related metabolic pathways (10–13). For example, Turmeric has shown significant potential in poultry nutrition, with its active compound, Curcumin, improving digestive enzyme activity, leading to better nutrient absorption and utilization. Additionally, Curcumin exhibits immunomodulatory and antioxidant properties, enhancing immune responses, reducing infections, and mitigating oxidative stress (14). Furthermore, *Astragalus* and ginseng polysaccharide supplementation have demonstrated significant benefits in improving growth performance, liver function, and intestinal villus morphology in weaned piglets. These plant polysaccharides improve nutrient absorption by promoting healthier intestinal structures and regulate immune function through activation of the TLR4-mediated MyD88-dependent signaling pathway (15).

The gut microbiome has recently gained significant attention for its vital role in regulating host health, immunity, and metabolism (16–20). In animals, the gut microbiota plays a key role in nutrient absorption, disease resistance, and overall productivity, drawing considerable interest from researchers aiming to enhance health and performance by modulating its composition and functions (21–23). Among ruminants, a large portion of the gut microbiome is housed in the rumen, a specialized digestive compartment. The rumen microbiome, distinct from the broader gut microbiome, is essential for breaking down plant fibers and fermenting nutrients into metabolites critical for energy production and growth (24).

Heat stress, however, can disrupt the microbial balance, impairing fermentation efficiency and overall digestive function. Investigating how dietary interventions influence rumen microbial composition and functions under heat stress is essential for developing targeted strategies to support microbial stability and enhance resilience in dairy cows. This study investigated a self-developed herbal formula as a dietary intervention to mitigate heat stress. By assessing its effects on physiological and biochemical attributes, antioxidant and immune responses, stress-related gene expression, and rumen microbial composition, this research evaluated whether the herbal formula could alleviate heat stress and support sustainable dairy farming practices.

## 2 Materials and methods

### 2.1 Animal ethics

All animal procedures used in this experiment were reviewed and approved by the Institutional Animal Care and Use Committee of Hebei Agricultural University (Baoding, China).

### 2.2 Herbal formula preparation

Herbal formula composition: Astragalus Root (Huangqi) 175 parts, Codonopsis Root (Dangshen) 60 parts, Angelica Sinensis (Danggui) 60 parts, Prepared Rehmannia Root (Shudi) 60 parts, Dried Tangerine Peel (Chenpi) 60 parts, Oriental Arborvitae Leaves (Cebaiye) 80 parts, Atractylodes Rhizome (Baizhu) 45 parts, Licorice Root (Gancao) 30 parts, Sichuan Lovage Rhizome (Chuanxiong) 30 parts, Ophiopogon Root (Maidong) 40 parts, White Peony Root (Baishao) 40 parts, Roasted Hawthorn Fruit (Zhishanzha) 100 parts, Roasted Radish Seeds (Laifuzi) 60 parts, Wine-processed Rhubarb Root (Jiu-Zhi Dahuang) 10 parts, Oriental Wormwood (Yincheng) 60 parts, Roasted Pig Hoof Shells (Zhi Zhutijia) 60 parts. All the components were purchased from Anguo Jufu Herbal Medicine Co., Ltd. (Hebei, China) and prepared following the guidelines of the classical Chinese Pharmacopoeia (State Pharmacopoeia Commission of the PRC, 2005).

### 2.3 Animals, experimental design and sample collection

This study was conducted on a commercial dairy farm in Hebei Province, China. A total of 198 lactating cows, with a parity distribution of 1 to 3 and an average milk production of 41.22 ± 5.05 kg/day, were selected for a 60-day experiment. The cows were divided into two groups: the Control group (*n* = 100), which received the farm standard total mixed ration (TMR), and the Herbs group (*n* = 98), which was fed the same TMR supplemented with the prepared herbal formula. The preliminary research has determined the optimal dosage of this self-developed traditional Chinese medicine formula for alleviating heat stress in dairy cows (25). Based on the findings, this study administered 20 g per head per day. The herbal formulation was evenly divided into three portions and thoroughly mixed into the TMR before each feeding, with each cow fed three times daily. Details of the TMR ingredients and nutritional composition are provided in Supplementary Table S1. Cows were housed in an open shed equipped with electric fans and provided with free access to water.

Ambient temperature (AT) and relative humidity (RH) in the cowshed were measured using KTH-350-I temperature and humidity data-logger (Kimo Industry Co., French) at 30-min intervals over 24 h for a 60-day experimental period to calculate the temperature-humidity index (THI) (26):


$$THI=\left(1.8\times AT+32\right)-\left[\left(0.55-0.0055\times RH\right)\times \left(1.8\times AT-26.8\right)\right]$$

Cows were fed three times daily at approximately 5:30 am, 1:30 pm, and 9:30 pm. Feed intake for each group was recorded on days 1–3, 14–16, 29–31, 44–46, and 58–60 of the experiment. On the same days, milk samples (100 mL) were collected from each group before feeding. The samples were then pooled in a 4:3:3 ratio (morning, midday, and evening milkings), stored at 4°C, and sent to the Dairy Herd Improvement (DHI) center (Shijiazhuang, China) within 12 h for composition analysis, including milk fat percentage (FP), protein percentage (PP), lactose percentage (LP), total solids (TS), somatic cell count (SCC), and blood urea nitrogen (BUN).

At the end of the experiment, blood samples were collected from 10 randomly selected cows in each group before the morning feeding. For each animal, two 5 mL blood samples were collected via the jugular vein: one into a vacuum tube without anticoagulant and the other into a tube containing heparin sodium. The sample in the anticoagulant-free tube was centrifuged at 3,000 g for 20 min at 4°C to obtain serum, which was stored at −20°C for subsequent analyses. Alanine aminotransferase (ALT), aspartate aminotransferase (AST), alkaline phosphatase (ALP), lactate dehydrogenase (LDH), creatine kinase (CK), blood urea nitrogen (BUN), total antioxidant capacity (T-AOC), superoxide dismutase (SOD), glutathione peroxidase (GSH-Px), and malondialdehyde (MDA) in serum were measured using a fully automatic biochemical analyzer (Gaomi Rainbow Analytical Instrument Co., Ltd., Shandong, China). Catalase (CAT) levels were determined using ELISA kit (Nanjing Jiancheng Biotechnology Research Institute Co., Ltd), following the manufacturer’s instructions. Interleukins (IL-1, IL-2, IL-6, IL-12), tumor necrosis factor-α (TNF-α), gamma-interferon (IFN-γ), and immunoglobulins (IgG, IgM, IgA) were analyzed using ELISA kit according to the manufacturer’s protocols (Dakemei Technology Co., Ltd., Beijing, China).

Blood samples collected in heparinized tubes were used to isolate peripheral blood lymphocytes (PBLCs) for the measurement of heat stress-related gene expression. Total RNA was extracted from PBLCs using a magnetic tissue/cell/blood total RNA kit (Tiangen Biotech, Beijing, China). Purified total RNA (1 μg) was reverse transcribed into cDNA using the PrimeScript^™^ RT Reagent Kit with gDNA Eraser (Takara Biotechnology, Dalian, China). RT-qPCR amplification was performed using SYBR^®^ Premix Ex Taq^™^ II (Tli RNase H Plus) (Takara Biotechnology, Dalian, China), according to the manufacturer’s protocol. Each gene was analyzed in three technical replicates. The relative changes in target gene expression were calculated using the $2^{-\mathrm{\Delta \Delta CT}}$ method. The RT-qPCR primers for each gene were designed using Primer Premier (version 5.0, Premier, Canada) and are listed in the Supplementary Table S2.

### 2.4 Data analysis

Statistical analysis was performed using an independent samples *t*-test in SPSS 18.0 software (SPSS, Chicago, IL, United States). The bar charts in this study display mean values with standard deviations, with significant differences between groups indicated by an asterisk (*) for *p* < 0.05.

### 2.5 Rumen microbial DNA extraction, PCR, and sequencing

Six cows from each group, previously used for blood collection, were selected for rumen fluid sampling via oral lavage. Approximately 10 mL of rumen content was collected from each cow and stored at −80°C for microbial sequencing. Genomic DNA from rumen content samples was extracted using the DNeasy PowerSoil Kit (Qiagen, Valencia, CA, United States) following the manufacturer’s protocol. DNA concentration and quality were assessed using a NanoDrop One spectrophotometer (Thermo Fisher Scientific, Madison, WI, United States) and 1% agarose gel electrophoresis. The DNA was subsequently diluted to the appropriate concentration for PCR.

The 16S rRNA gene regions of bacteria and archaea were amplified using specific primers. For the bacterial 16S V3–V4 region, the forward primer (ACTCCTACGGGAGGCAGCAG) and reverse primer (GGACTACHVGGGTWTCTAAT) were used, while for the archaeal 16S V3–V4 region, the forward primer (ACGGGGYGCAGCAGGCGCGA) and reverse primer (GGACTACVSGGGTATCTAAT) were employed. PCR conditions for bacteria included an initial denaturation at 94°C for 5 min, followed by 28 cycles of denaturation at 94°C for 30 s, annealing at 55°C for 30 s, and extension at 72°C for 60 s, with a final extension at 72°C for 7 min. For archaea, PCR conditions included an initial denaturation at 94°C for 5 min, followed by 35 cycles of denaturation at 94°C for 30 s, annealing at 63°C for 30 s, and extension at 72°C for 60 s, with a final extension at 72°C for 7 min. All reactions were held at 4°C after completion.

The PCR products were electrophoresed on a 1% agarose gel to confirm the expected size of the amplicons. Purification of the PCR products was performed using Agencourt AMPure XP beads, and the purified products were used to create libraries, which were sequenced on the Illumina MiseqPE300 platform (AllweGene Technology Company, Beijing, China).

### 2.6 Microbiome data analysis

The 16S rRNA gene sequences for bacteria and archaea were analyzed using QIIME 2 (2019.7 release) (27). Paired-end reads were imported into QIIME 2, merged to combine forward and reverse reads, and filtered to exclude low-quality sequences based on quality scores. The Deblur plugin was used to denoise sequences, remove chimeric and low-abundance reads, and generate amplicon sequence variants (ASVs) with 100% sequence identity, providing high-resolution insights into microbial communities. The ASVs, along with their read counts per sample, were used to construct an ASV table, which was subsequently rarefied to standardize sequencing depth across all samples for diversity analyses. Representative sequences derived from the ASVs were used for taxonomic classification (28). Taxonomic classification was achieved using a naive Bayes classifier trained on the Greengenes database (version 13_8) tailored to the primer region used in this study (29).

Alpha and beta diversity metrics were calculated using rarefied data to standardize sequencing depth across all samples, reducing biases from differences in sequencing effort. In this study, alpha diversity metrics, including Shannon, Observed_ASVs, and Chao1 indices, and beta diversity metrics, including Bray–Curtis and Jaccard distances, were assessed. Statistical significance of beta diversity differences between groups was evaluated using ANOSIM. Functional prediction was performed using PICRUSt2, which generated KEGG pathway-based outputs to infer the functional potential of the microbial communities (30, 31).

Differences in alpha diversity between the two groups were assessed using the Kruskal–Wallis test in R to evaluate statistical significance. Principal coordinate analysis (PCoA) plots were used to visualize Bray–Curtis and Jaccard distances. Stacked bar plots of microbial composition at the phylum, family, and genus levels were created in R using the ggplot2 package (32). Differentially abundant taxa between the two groups were identified using the LEfSe algorithm on the Galaxy platform^1^ with default settings (LDA score >2). The importance of ASVs in differentiating the control and Herbs groups was evaluated using the randomForest package in R. Random Forest classification models were built with 1,000 trees to calculate the importance of each ASV based on the mean decrease in accuracy. The varImpPlot function was used to visualize the top 20 ASVs with the highest importance values, and the pheatmap package was employed to display the relative abundance of these ASVs across the two groups (33). Functional predictions based on PICRUSt2 results were visualized using STAMP (Statistical Analysis of Metagenomic Profiles) software (34).

## 3 Results

### 3.1 Herbal formula improved feed intake, milk production, antioxidant capacity, and immune responses in heat-stressed dairy cows

**Figure 1:**
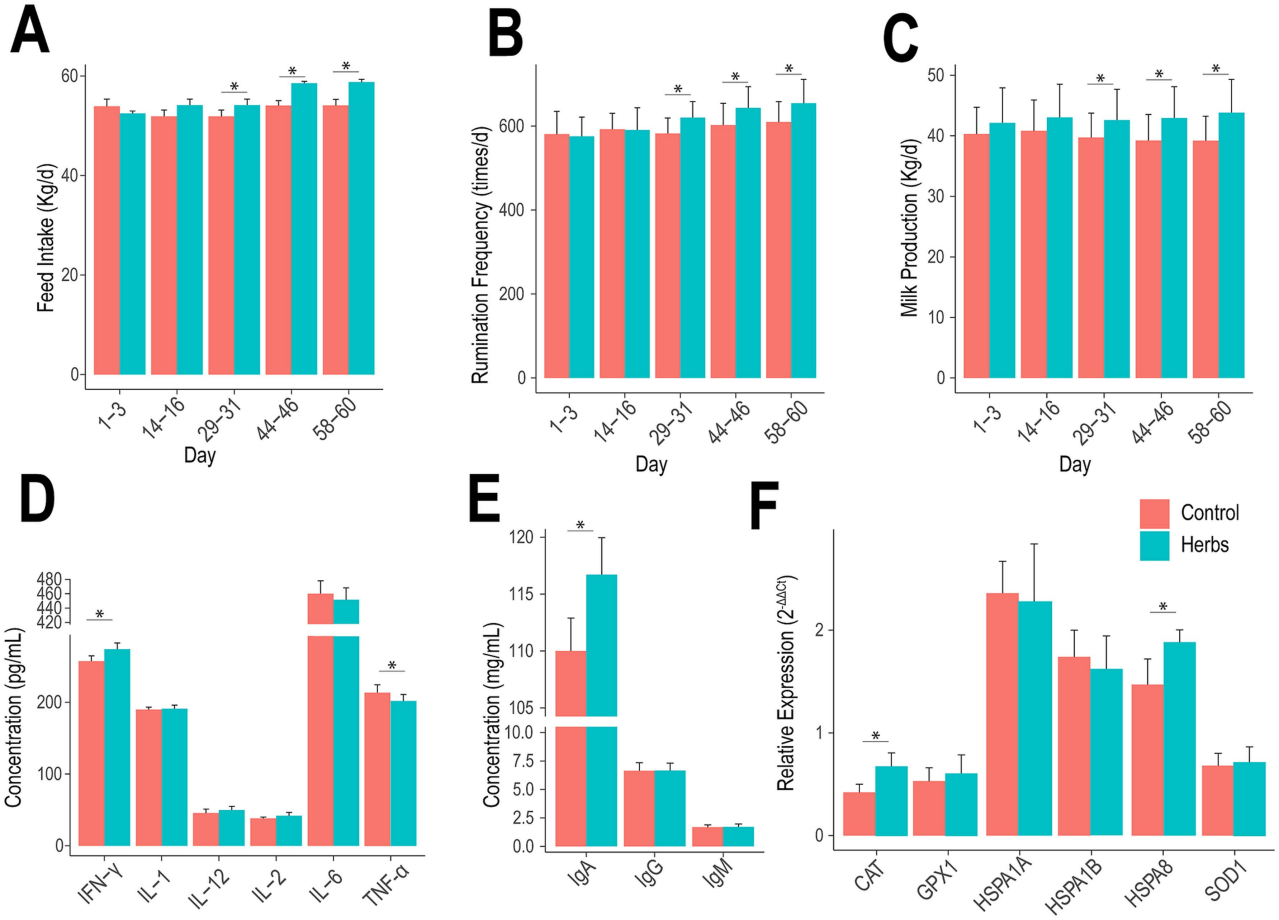
Effects of herbal formula supplementation on feed intake **(A)**, rumination frequency **(B)**, milk production **(C)**, cytokine levels **(D)**, immunoglobulin levels **(E)**, and relative expression of heat stress-related genes **(F)**.

The diurnal temperature in the experimental barn averaged 26.51°C, with an average relative humidity of 71.65% and an average temperature-humidity index of 75.71 throughout the experimental period, indicating potential heat stress in the animals (Supplementary Figure S1). The Herbs group showed significant improvements in feed intake, rumination frequency, and milk production compared to the Control group on days 29–31, 44–46, and 58–60 (*p* < 0.05; Figures 1A–C), with no significant differences observed on days 1–3 and 14–16 (*p* > 0.05). Similarly, the effects of the herbal formula on milk composition were primarily evident at these later time points (29–31 days, 44–46 days, and 58–60 days; Table 1). Compared to the Control group, the Herbs group showed significantly higher FP, PP, LP, and TS at these time points (*p* < 0.05), except for TS at 29–31 days and PP at 44–46 days, where no significant differences were observed (*p* > 0.05). Additionally, SCC and BUN levels were consistently reduced in the Herbs group at these three later time points (*p* < 0.05). Together, these findings demonstrate that the effects of herbal formula become more pronounced over time, resulting in significant improvements in milk yield, milk quality, and health indicators in heat-stressed dairy cows.

**Table 1:** Effects of the herbal formula on milk composition in heat-stressed dairy cows.

Day	Groups	FP (%)	PP (%)	LP (%)	TS (%)	SCC (w/mL)	BUN (mg/dL)
1–3 days	Control	4.02 ± 0.06^a^	3.17 ± 0.04^a^	4.85 ± 0.09^a^	12.35 ± 0.17^a^	18.29 ± 0.99^a^	9.87 ± 1.17^a^
	Herbs	4.02 ± 0.09^a^	3.20 ± 0.03^a^	4.83 ± 0.07^a^	12.24 ± 0.38^a^	18.30 ± 0.97^a^	10.93 ± 1.79^a^
14–16 days	Control	3.91 ± 0.05^a^	3.10 ± 0.04^a^	4.47 ± 0.06^a^	12.17 ± 0.70^a^	17.77 ± 0.38^a^	11.63 ± 1.53^a^
	Herbs	3.96 ± 0.04^a^	3.18 ± 0.06^a^	4.42 ± 0.07^a^	12.33 ± 0.19^a^	17.37 ± 0.15^a^	11.73 ± 1.08^a^
29–31 days	Control	3.73 ± 0.07^b^	3.02 ± 0.08^b^	4.54 ± 0.09^b^	11.99 ± 0.28^a^	18.47 ± 0.38^a^	12.53 ± 1.86^a^
	Herbs	4.03 ± 0.01^a^	3.18 ± 0.06^a^	4.96 ± 0.07^a^	12.07 ± 0.29^a^	16.83 ± 0.35^b^	11.03 ± 0.50^b^
44–46 days	Control	3.83 ± 0.10^b^	3.08 ± 0.01^a^	4.51 ± 0.19^b^	11.94 ± 0.20^b^	18.73 ± 0.15^a^	12.27 ± 0.86^a^
	Herbs	3.90 ± 0.02^a^	3.11 ± 0.03^a^	4.80 ± 0.04^a^	12.26 ± 0.06^a^	16.97 ± 0.76^b^	10.23 ± 0.42^b^
58–60 days	Control	3.77 ± 0.04^b^	3.00 ± 0.06^b^	4.75 ± 0.08^b^	11.22 ± 1.26^b^	18.50 ± 0.85^a^	12.83 ± 1.70^a^
	Herbs	3.95 ± 0.03^a^	3.12 ± 0.05^a^	4.92 ± 0.09^a^	11.93 ± 1.01^a^	16.67 ± 0.15^b^	9.00 ± 1.97^b^

FP, fat percentage; PP, protein percentage; LP, lactose percentage; TS, total solids; SCC, somatic cell count; BUN, blood urea nitrogen. For each time point, data with different superscripts within the same column indicate significant differences (*p* < 0.05).

The administration of herbal formula significantly affected serum biochemical parameters in heat-stressed dairy cows (Table 2). Serum BUN levels were significantly reduced in the herbs group (*p* < 0.05), consistent with the previously observed decrease in milk BUN levels. Additionally, the herbs group exhibited significantly lower MDA levels and higher T-AOC, SOD, GSH-Px, and CAT activities (*p* < 0.05), indicating enhanced antioxidant defenses and reduced oxidative stress. No significant differences were observed in ALT, AST, ALP, LDH, or CK levels between the two groups (*p* > 0.05).

**Table 2:** Effects of the herbal formula on serum biochemical parameters in heat-stressed dairy cows.

Parameter	Control	Herbs
ALT (U/L)	20.45 ± 3.20^a^	19.43 ± 2.59^a^
AST (U/L)	73.32 ± 7.58^a^	71.65 ± 16.87^a^
ALP (U/L)	47.75 ± 1.31^a^	48.50 ± 3.92^a^
LDH (U/L)	553.40 ± 9.08^a^	548.20 ± 17.72^a^
CK (U/L)	116.33 ± 8.88^a^	115.11 ± 7.05^a^
BUN (mmol/L)	4.49 ± 0.31^a^	3.83 ± 0.32^b^
T-AOC (mmol/L)	12.46 ± 0.95^b^	13.50 ± 0.60^a^
SOD (mmol/L)	51.61 ± 2.54^b^	53.71 ± 1.89^a^
GSH-Px (μmol/L)	297.77 ± 14.64^b^	328.65 ± 12.43^a^
MDA (nmol/mL)	2.74 ± 0.87^a^	1.87 ± 0.55^b^
CAT (IU/L)	8.08 ± 0.69^b^	8.98 ± 0.86^a^

ALT, alanine aminotransferase; AST, aspartate aminotransferase; ALP, alkaline phosphatase; LDH, lactate dehydrogenase; CK, creatine kinase; BUN, blood urea nitrogen; T-AOC, total antioxidant capacity; SOD, superoxide dismutase; GSH-Px, glutathione peroxidase; MDA, malondialdehyde; AT, catalase. Data with different superscripts in the same row indicates significant difference (*p* < 0.05).

The pro-inflammatory cytokine analysis showed that the Herbs group had significantly higher levels of IFN-γ and lower levels of TNF-α compared to the Control group (*p* < 0.05), with no significant differences observed for IL-1, IL-2, IL-6, and IL-12 (*p* > 0.05; Figure 1D). Immunoglobulin analysis revealed significantly higher IgA concentrations in the Herbs group (*p* < 0.05), while IgG and IgM concentrations remained similar between the two groups (*p* > 0.05; Figure 1E). For heat stress-related gene expression, the Herbs group displayed significantly higher expression of *CAT* and *HSPA8* (*p* < 0.05), whereas no significant differences were detected for *GPX1*, *HSPA1A*, *HSPA1B*, and *SOD1* (*p* > 0.05; Figure 1F).

### 3.2 Herbal formula significantly altered rumen bacterial and archaeal community structures

Alpha diversity was assessed using the Shannon index, observed ASVs, and Chao1 index for both bacterial (Supplementary Figure S2A) and archaeal (Supplementary Figure S2B) communities. In the bacterial community, the Herbs group exhibited slightly lower Shannon index and observed ASVs compared to the Control group, while the Chao1 index was slightly higher. For the archaeal community, the Herbs group showed slightly lower values for all three indices compared to the Control group. However, statistical analysis revealed that none of these differences were significant (*p* > 0.05).

**Figure 2:**
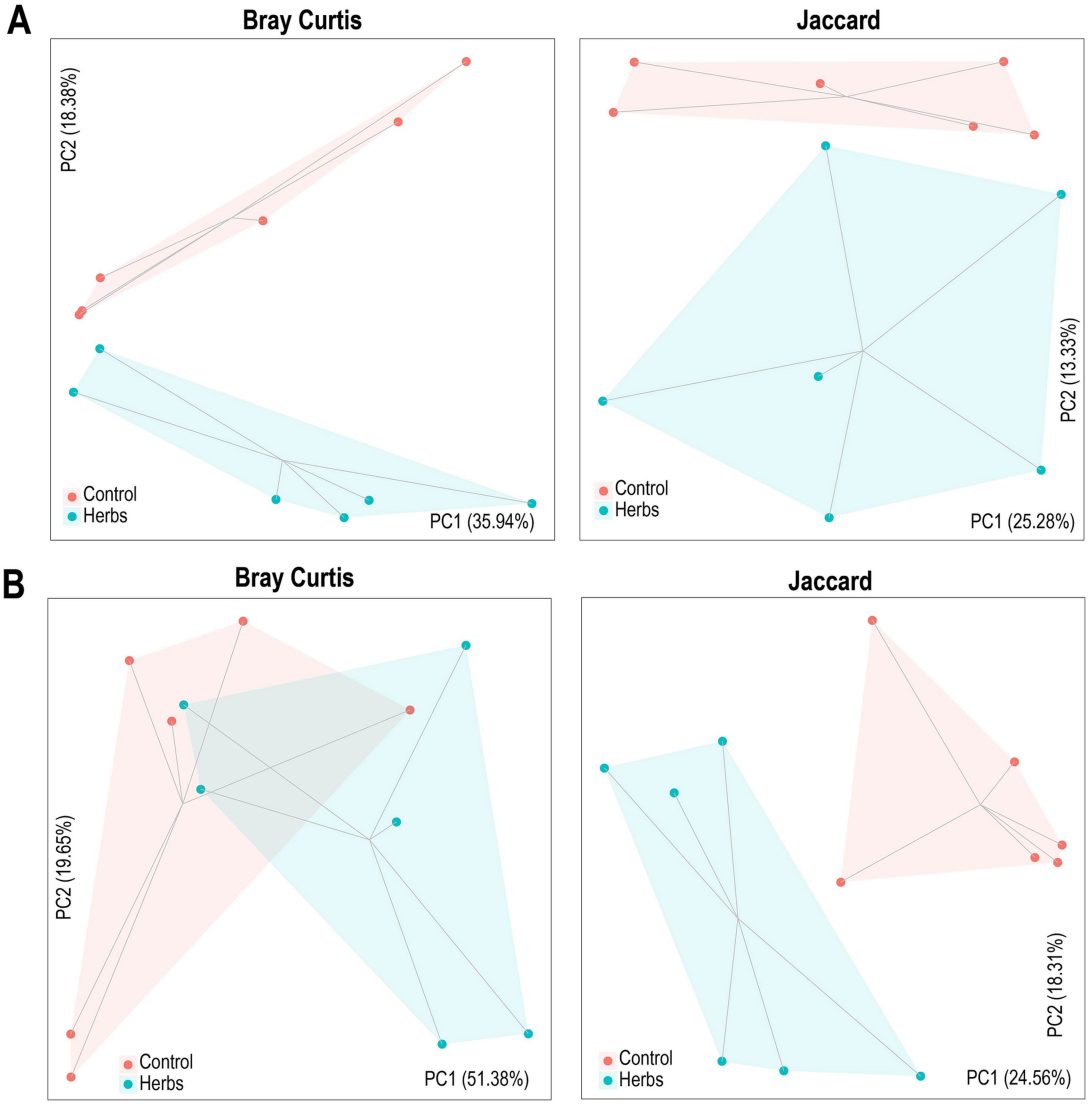
Principal coordinate analysis (PCoA) of Bray–Curtis and Jaccard distances for bacterial **(A)** and archaeal **(B)** communities. Each point represents a unique sample, with shaded areas indicating group clustering. Lines connect samples within each group, and statistical differences were evaluated using ANOSIM.

The PCoA plots illustrate differences in community structure between the Control and Herbs groups, supported by ANOSIM results. For bacteria (Figure 2A), Bray–Curtis distance showed significant differences (*R* = 0.22, *p* = 0.04), while Jaccard distance did not show significant separation (*R* = 0.09, *p* = 0.19). For archaea (Figure 2B), both Bray–Curtis (*R* = 0.22, *p* = 0.04) and Jaccard distances (*R* = 0.49, *p* = 0.004) indicated significant differences.

**Figure 3:**
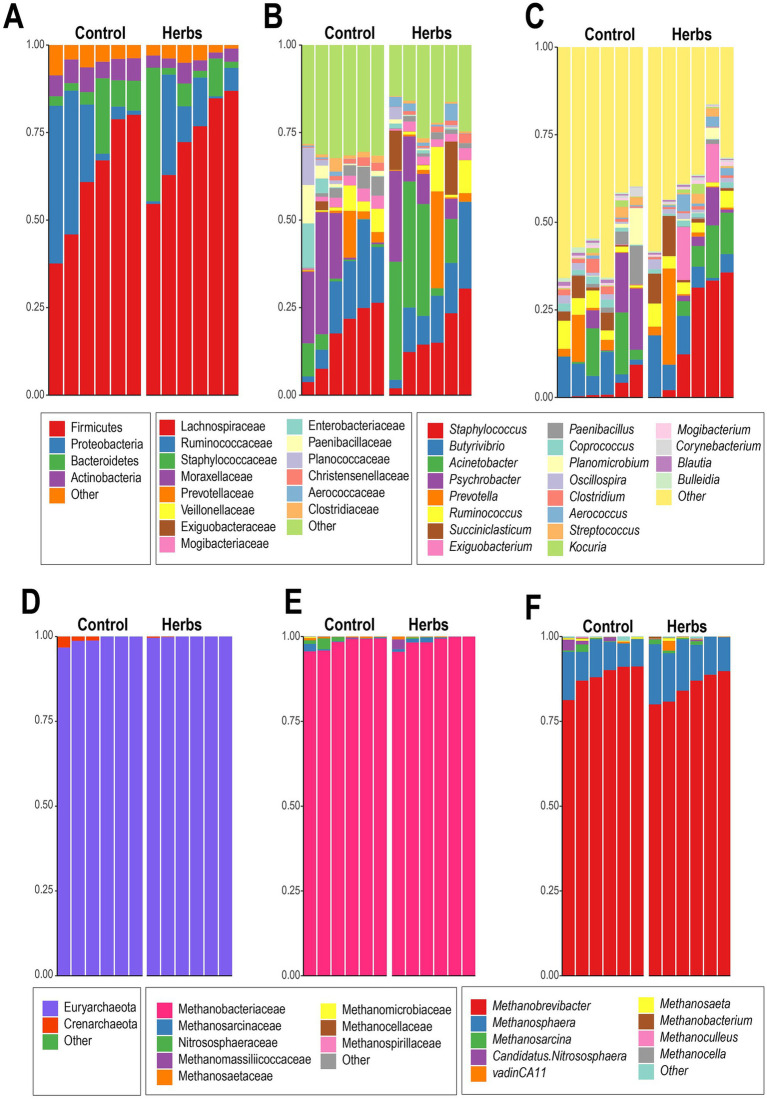
Taxonomic composition of bacterial and archaeal communities in the Control and Herbs groups. Bacterial composition at the phylum level **(A)**, family level **(B)**, and genus level **(C)**. Archaeal composition at the phylum level **(D)**, family level **(E)**, and genus level **(F)**.

For the rumen bacteria, Firmicutes and Proteobacteria were the dominant phyla across all samples, accounting for 73.0 and 10.1% of the sequences in the Herbs group, compared to 61.7 and 19.1% in the Control group, respectively (Figure 3A). The relative abundance of Bacteroidetes slightly increased in the Herbs group (10.2%) compared to the Control group (7.7%), while Actinobacteria decreased (Control: 6.1%, Herbs: 3.4%). At the family level (Figure 3B), Lachnospiraceae and Ruminococcaceae were consistently dominant, with a slight decrease in Lachnospiraceae from 17.0% (Control) to 16.2% (Herbs) and stable levels of Ruminococcaceae around 13%. Staphylococcaceae, however, exhibited an increase in the Herbs group (Control: 2.6%, Herbs: 19.4%). At the genus level (Figure 3C), *Staphylococcus* exhibited the most pronounced change, increasing significantly in the Herbs group (19.1%) compared to the Control group (2.5%) (Figure 3C). Other genera showed more variable and inconsistent patterns between groups, with no clear trends emerging.

The archaeal community was dominated by Euryarchaeota, accounting for around 99% of the sequences in both the Control and Herbs groups (Figure 3D). At the family level, Methanobacteriaceae was the predominant family, representing approximately 98% of the sequences in both groups (Figure 3E). At the genus level, the community was primarily composed of *Methanobrevibacter*, which accounted for 88.1% in the Control group and 85.0% in the Herbs group, and *Methanosphaera*, contributing 9.5 and 13.2% in the Control and Herbs groups, respectively (Figure 3F). These results indicated that the archaeal community was dominated by similar taxa in both groups, although beta diversity analysis revealed differences in community structure.

### 3.3 Distinct taxa represented in Control and Herbs groups

**Figure 4:**
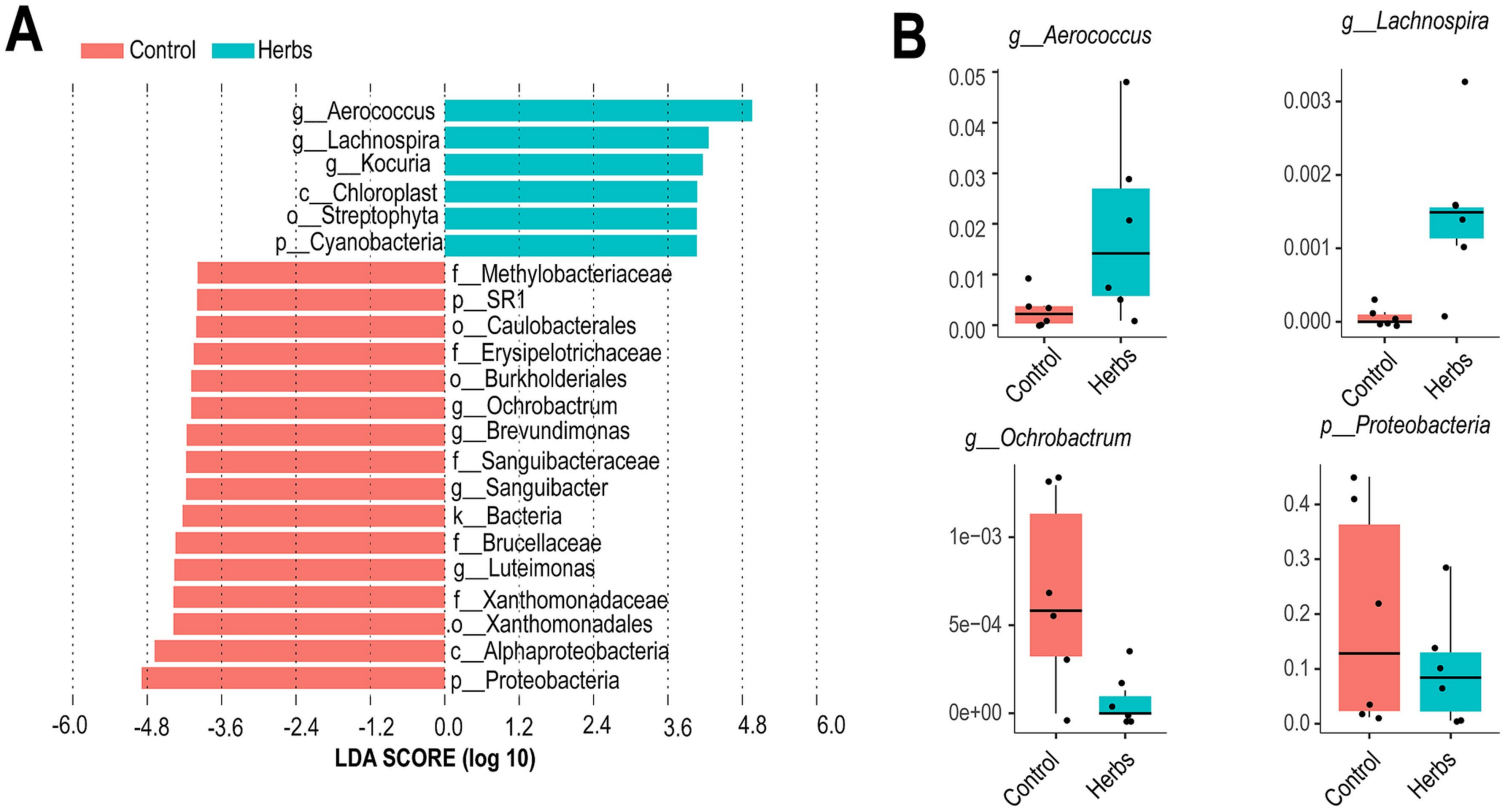
Differentially abundant taxa between the Control and Herbs groups identified by LEfSe analysis. Linear discriminant analysis (LDA) scores show the taxa enriched in the Control (red) and Herbs (blue) groups **(A)**. Relative abundances of selected taxa in the two groups **(B)**.

LEfSe analysis revealed distinct taxa enriched in the Control and Herbs groups (Figure 4A). The Herbs group showed significant enrichment of *Aerococcus*, *Lachnospira*, and *Kocuria*, along with taxa associated with Chloroplast and Streptophyta (*p* < 0.05). In contrast, the Control group had higher abundances of *Ochrobactrum*, Sanguibacteraceae, and Proteobacteria (*p* < 0.05). The relative abundances of *Aerococcus*, *Lachnospira*, *Ochrobactrum*, and Proteobacteria were further displayed in boxplots, highlighting the greater abundance of *Aerococcus* and *Lachnospira* in the Herbs group and *Ochrobactrum* and Proteobacteria in the Control group (Figure 4B).

**Figure 5:**
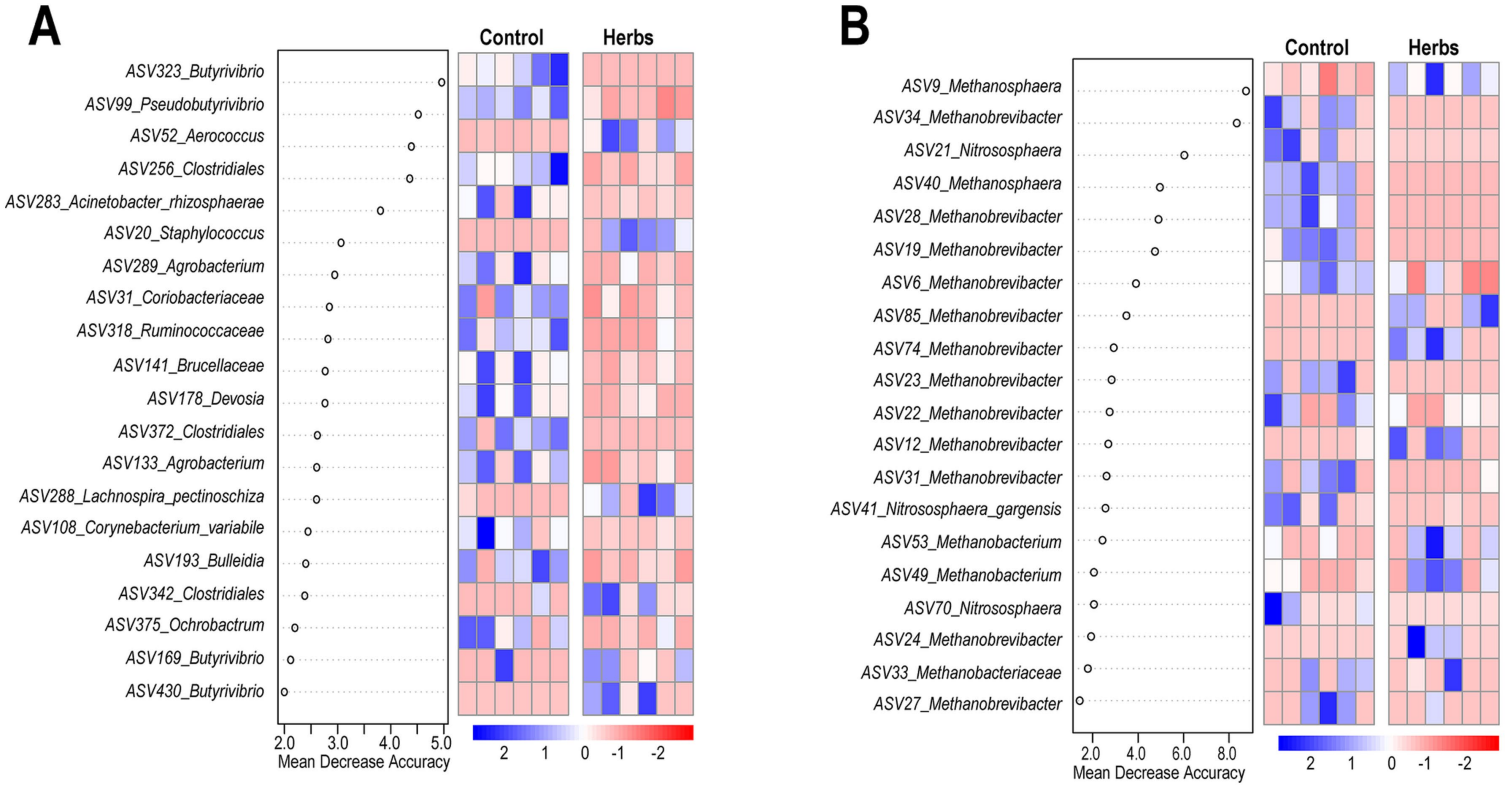
Gut microbiota signature of the Control and Herbs groups determined by Random Forest. Top 20 ASVs from the bacterial community that best differentiate the Herbs group from the Control group **(A)**. Top 20 ASVs from the archaeal community **(B)**. Heatmaps display the relative abundance (log10 transformed) of ASVs selected by Random Forest, with blue and red indicating higher and lower abundance, respectively.

The random forest analysis and heatmap visualization identified key ASVs that best differentiate the Control and Herbs groups. Among the top 20 ASVs identified by random forest analysis, bacterial taxa included *Butyrivibrio*, *Aerococcus*, and *Staphylococcus* (Figure 5A), while archaeal taxa included *Methanosphaera*, *Methanobrevibacter*, and *Nitrososphaera* (Figure 5B).

### 3.4 Herbal formula modulated functional pathways in rumen bacterial and archaeal communities

**Figure 6:**
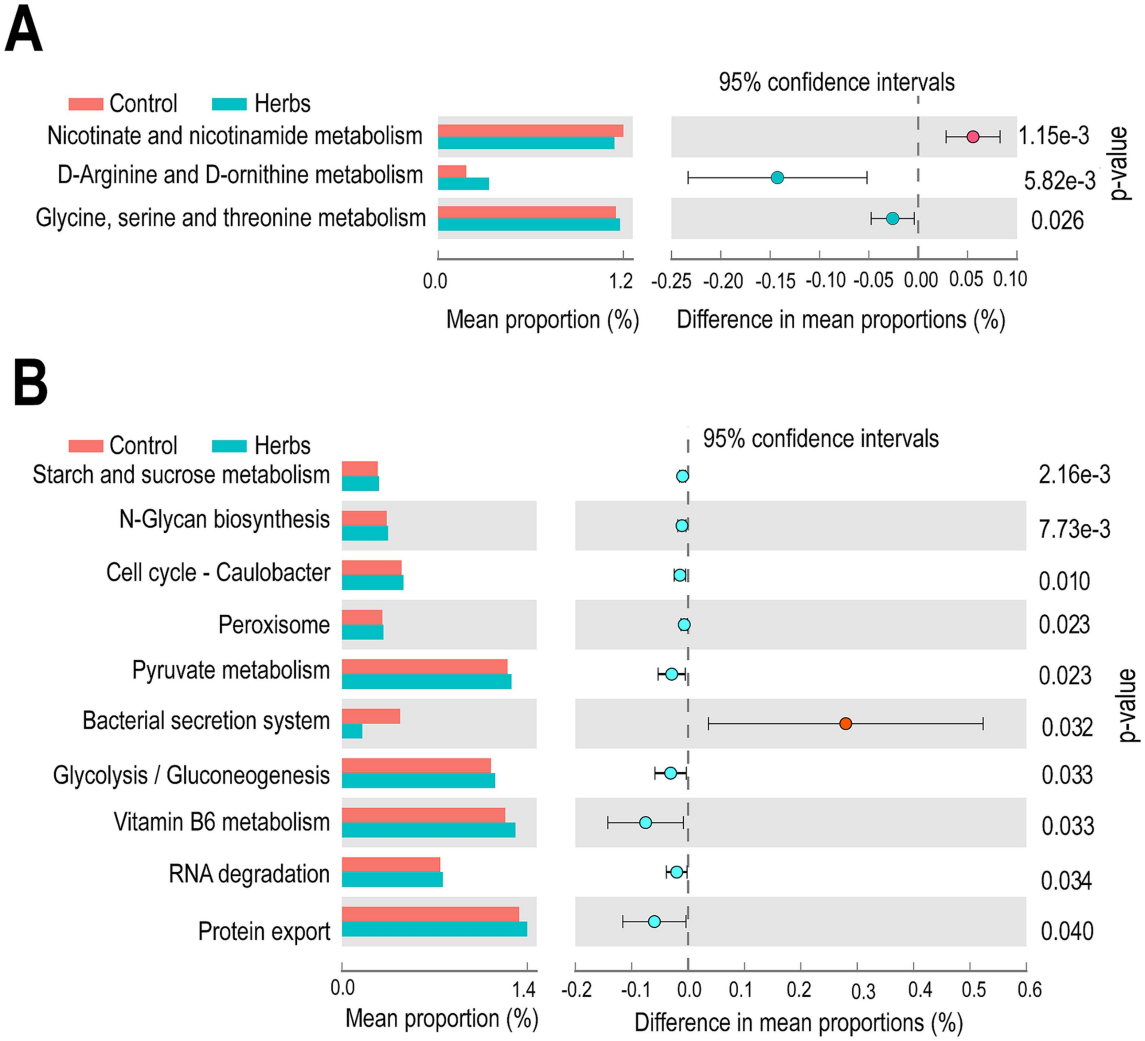
Predicted KEGG functional pathways of bacterial **(A)** and archaeal **(B)** communities in the Control (red) and Herbs (blue) groups. The third-level KEGG pathways are shown, with mean proportions and 95% confidence intervals. Significant differences between the groups were determined using a *t*-test (*p* < 0.05).

The PICRUSt analysis revealed significant differences (*p* < 0.05) in predicted KEGG pathways between the Control and Herbs groups in both bacterial (Figure 6A) and archaeal (Figure 6B) communities. In the bacterial community (Figure 6A), genes related to nicotinate and nicotinamide metabolism were predicted to be more abundant in the Control group, while genes involved in D-arginine and D-ornithine metabolism and glycine, serine, and threonine metabolism were enriched in the Herbs group. In the archaeal community (Figure 6B), 10 pathways were identified as significantly different between groups (*p* < 0.05), with nine pathways, including those involved in starch and sucrose metabolism, pyruvate metabolism and glycolysis/gluconeogenesis, predicted to be enriched in the Herbs group. Only one pathway, bacterial secretion system, was predicted to be more abundant in the Control group. These results suggested that the herbal formula influenced key metabolic pathways in both bacterial and archaeal communities.

## 4 Discussion

Heat stress is a significant challenge in the livestock industry, particularly for dairy cattle, due to its impact on both animal welfare and economic productivity. As global temperatures rise and greenhouse gas emissions increase, the impact of heat stress is expected to become more severe, posing ongoing challenges for dairy farming (1). Dairy cattle are especially vulnerable to heat stress because of their high metabolic heat production associated with milk synthesis, making them less able to cope with elevated temperatures compared to other livestock species (35). This susceptibility not only reduces milk production but also impacts overall health and fertility, leading to substantial economic losses (2).

Heat stress is known to induce oxidative stress by generating excessive reactive oxygen species (ROS), which can damage tissues and cells (36). Antioxidant enzymes, such as CAT, SOD, GSH-Px, and T-AOC, play a critical role in scavenging ROS, enhancing the body antioxidant defenses, and maintaining overall health (37, 38). In this study, supplementation with herbal formula significantly enhanced the activities of these enzymes, which likely contributed to the observed reduction in serum MDA levels, a key marker of oxidative damage (39). In addition, heat stress is well-documented to elevate BUN levels, indicative of disrupted protein metabolism (40, 41). Notably, herbal formula supplementation significantly reduced BUN levels in both serum and milk under heat-stress conditions (*p* < 0.05), suggesting an improvement in protein metabolism.

Herbal formula supplementation significantly upregulated the expression of the *HSPA8* gene (*p* < 0.05), a key member of the heat shock protein family that is critical for cellular stress responses. Heat shock proteins like *HSPA8* stabilize proteins as intra-cellular chaperones, prevent aggregation, and assist in protein refolding, which are essential functions under heat stress conditions (42–44). The increased expression of *HSPA8* observed in the herbs group suggested that our self-developed herbal formula enhanced the cell ability to cope with heat-induced protein damage. This upregulation likely strengthened cellular resilience, mitigating the impact of heat stress on cellular function and viability. In addition, several studies have demonstrated the pivotal role of *HSPA8* in milk protein synthesis in cows (45, 46). In this study, PP showed significantly increased in Herbs group (*p* < 0.05), it may be related to the significant increase in *HSPA8* expression level.

In this study, the Herbs group showed significantly higher levels of IgA (*p* < 0.05), an important serum immunoglobulin that mediates various protective functions through interactions with specific receptors and immune mediators (47). Elevated IgA levels may further contribute to maintaining overall health and mitigating the adverse effects of heat stress by strengthening the immune system and providing additional protection against stress-related vulnerabilities. The Herbs group also exhibited significantly reduced levels of TNF-α (*p* < 0.05), a pro-inflammatory cytokine. Lower TNF-α levels suggested that the herbal formula may help alleviate systemic inflammation and support immune regulation under heat stress. Together, these findings demonstrated the effectiveness of herbal formula in mitigating heat stress by enhancing antioxidant defenses, improving immune responses, reducing oxidative damage, supporting metabolic function, and reducing systemic inflammation.

The beneficial effects of herbal formula on heat-stressed cows may be attributed to its key components, such as Astragalus (Huangqi), which has been widely recognized for its antioxidant, immune-enhancing, and anti-inflammatory properties (48). Its bioactive components, such as polysaccharides and saponins, have been shown to scavenge free radicals, enhance the activities of antioxidant enzymes like CAT and SOD, and reduce oxidative stress in livestock (48, 49). Astragalus polysaccharides are also known for their immune-modulatory properties, which may have contributed to the elevated IgA levels observed in the Herbs group. Furthermore, Astragalus calycosin exhibits significant anti-inflammatory activity by modulating inflammatory pathways, such as the NF-κB signaling pathway, and reducing the production of pro-inflammatory cytokines, including TNF-α (48, 50).

Beyond its benefits in mitigating oxidative damage and supporting immune regulation, herbal formula significantly impacted the rumen microbiome, a key player in nutrient digestion and absorption (51). Notably, this self-developed herbal formula reduced the relative abundance of Proteobacteria, a phylum increasingly recognized as a microbial marker of disease. Increased relative abundance of Proteobacteria has been associated with inflammation-driven diseases such as metabolic disorders, inflammatory bowel disease, and respiratory illnesses (52). In our study, the reduction of Proteobacteria in the Herbs group suggested that herbal formula may contribute to a healthier rumen microbial environment, potentially reducing the risk of inflammation. At the genus level, the rumen microbiome composition in the Control group showed considerable variability between samples, whereas the Herbs group exhibited a relatively more stable composition, with *Staphylococcus* consistently emerging as the dominant genus. Although *Staphylococcus* is generally not a major or abundant member of the rumen microbiome (53–56), herbal formula appeared to create favorable conditions for its proliferation within the rumen environment. While some members of *Staphylococcus*, such as *S. aureus*, are associated with diseases like mastitis in cattle (57, 58), others play beneficial roles. For example, *S. xylosus* contributes to lactic acid fermentation (59), *S. carnosus* has nitrate-reducing and antimicrobial properties (60), and *S. chromogenes* may inhibit pathogenic colonization through microbial competition (61). Although the specific *Staphylococcus* species in the Herbs group could not be identified, their increased abundance likely contributed to a more balanced and efficient microbial ecosystem, coinciding with the improved performance and health observed in Herbs group.

Other genera with increased abundance in the Herbs group included *Aerococcus* and *Lachnospira*. *Aerococcus* is a genus of Gram-positive bacteria primarily studied in human, where certain species are recognized as opportunistic pathogens (62–64). However, its role within the rumen microbiome remains largely unexplored. Its increased presence in Herbs group may reflect a microbial response to herbal formula, though its specific role in rumen environment warrants further investigation. In contrast, *Lachnospira* is well-known for its ability to ferment dietary fibers into short-chain fatty acids (SCFAs) such as acetate, propionate, and butyrate (65). These SCFAs are critical for maintaining gut health, regulating immune responses, promoting anti-inflammation, and supporting metabolic health (66, 67). The higher abundance of *Lachnospira* in the Herbs group could suggest enhanced fiber degradation, potentially improving energy utilization. Additionally, *Ochrobactrum*, a genus that includes opportunistic pathogens (68), was significantly reduced in the Herbs group. This reduction, along with changes in other microbial populations, highlighted herbal formula potential to improve microbial balance and support overall health.

The PICRUSt analysis revealed distinct differences in the predicted functional potential of metabolic pathways between the Herbs and Control groups, with a notably greater number of pathways altered in archaea compared to bacteria. However, it should be noted that PICRUSt-based functional predictions derive potential metabolic pathways from 16S rRNA gene data rather than whole metagenomes. One of the most notable changes among the archaeal pathways was the significant reduction in the bacterial secretion system in Herbs group. This system is essential for transporting proteins and mediating interactions between microbes and their environment or host. In bacterial pathogens, secretion systems are essential for virulence, facilitating diverse functions such as enhancing bacterial adhesion, scavenging environmental resources, and directly disrupting host cell functions (69). However, their role in archaea remains unclear, and the observed reduction may reflect broader shifts in microbial interactions or metabolic adaptation rather than pathogenic mechanisms. Additionally, pathways related to glycolysis/gluconeogenesis and pyruvate metabolism in archaea were significantly enriched in the Herbs group. These pathways are fundamental to energy production and carbon cycling in the rumen, suggesting that herbal formula enhanced microbial energy utilization and fermentation efficiency. This was further supported by the observed enrichment in the starch and sucrose metabolism pathway, reflecting an increased capacity for carbohydrate degradation.

## 5 Conclusion

This study showed that our self-developed herbal formula enhanced antioxidant capacity, strengthened immune responses, and improved cellular resilience in dairy cows under heat stress. Additionally, they helped mitigate heat stress by regulating the composition, function, and metabolites of the rumen microbiota, ultimately improving overall health. These findings highlighted their potential as a natural dietary strategy for alleviating heat stress in dairy cows.

## Data Availability

Raw data were submitted to the National Center for Biotechnology Information (NCBI) Short Read Archive database and are available with BioProject accession number PRJNA1225285 (https://www.ncbi.nlm.nih.gov/bioproject/PRJNA1225285/).

## Footnotes

^1^ http://mbac.gmu.edu:8080/

## Supplementary material

SUPPLEMENTARY FIGURE S1 Dynamics of the thermal environment in dairy barns. The temperature unit is °C and the relative humidity unit is %. Cows suffer from heat stress almost 24 h a day.

SUPPLEMENTARY FIGURE S2 Comparison of alpha diversity metrics between the Control and Herbs groups for bacterial **(A)** and archaeal **(B)** communities. Diversity indices include the Shannon index, observed ASVs, and Chao1 index, illustrating microbial diversity and richness. No statistically significant differences were detected between the two groups.
