# Supplementary figures and images for: Herbal formula alleviates heat stress by improving physiological and biochemical attributes and modulating the rumen microbiome in dairy cows
Source: Front Vet Sci. 2025 Mar 7;12:1558856. doi: 10.3389/fvets.2025.1558856 (PMC11925914; doi:10.3389/fvets.2025.1558856)

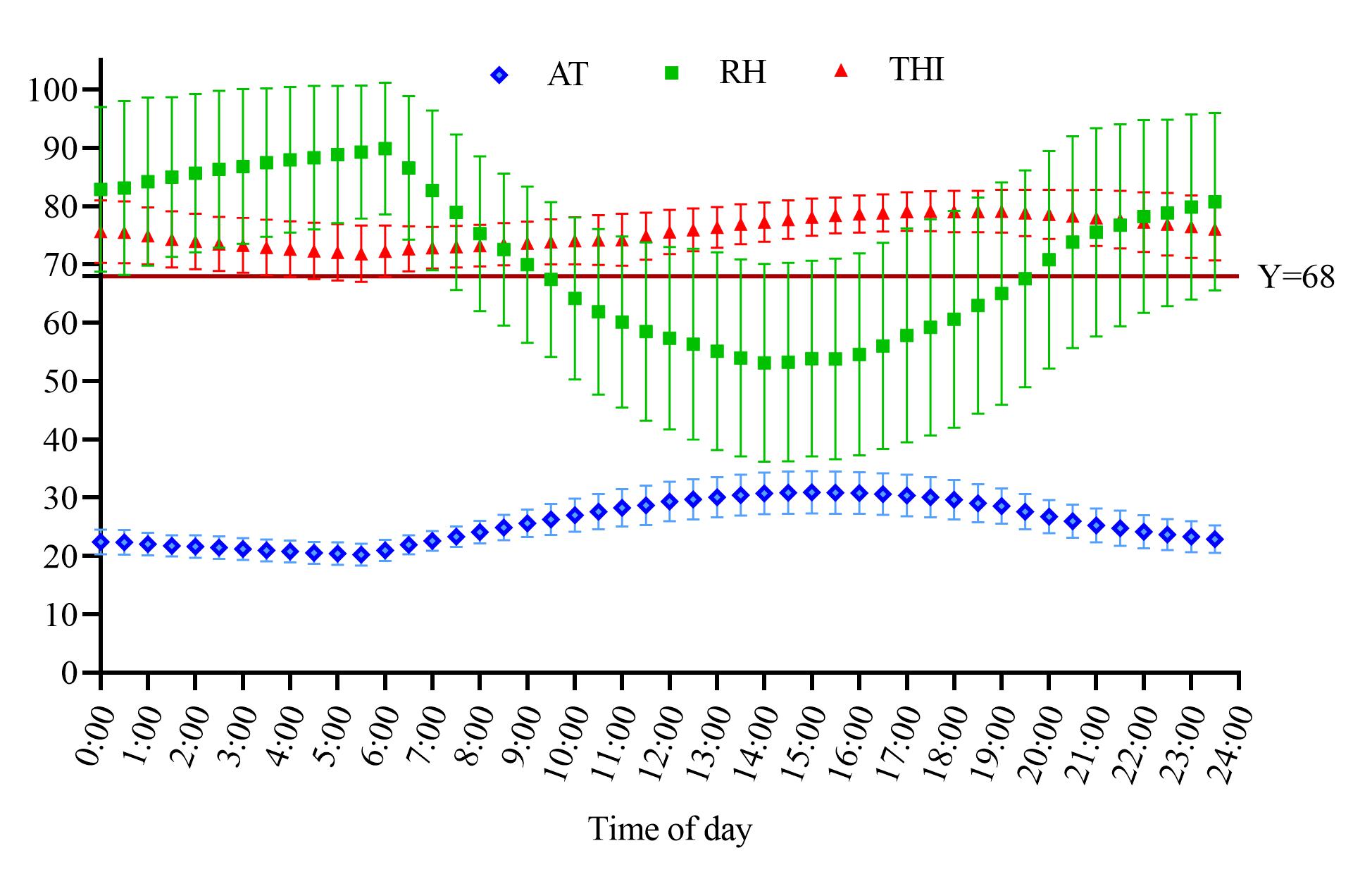

Supplement: SUPPLEMENTARY FIGURE S1 — Dynamics of the thermal environment in dairy barns. The temperature unit is °C and the relative humidity unit is %. Cows suffer from heat stress almost 24 h a day. [file Image_1.jpeg]

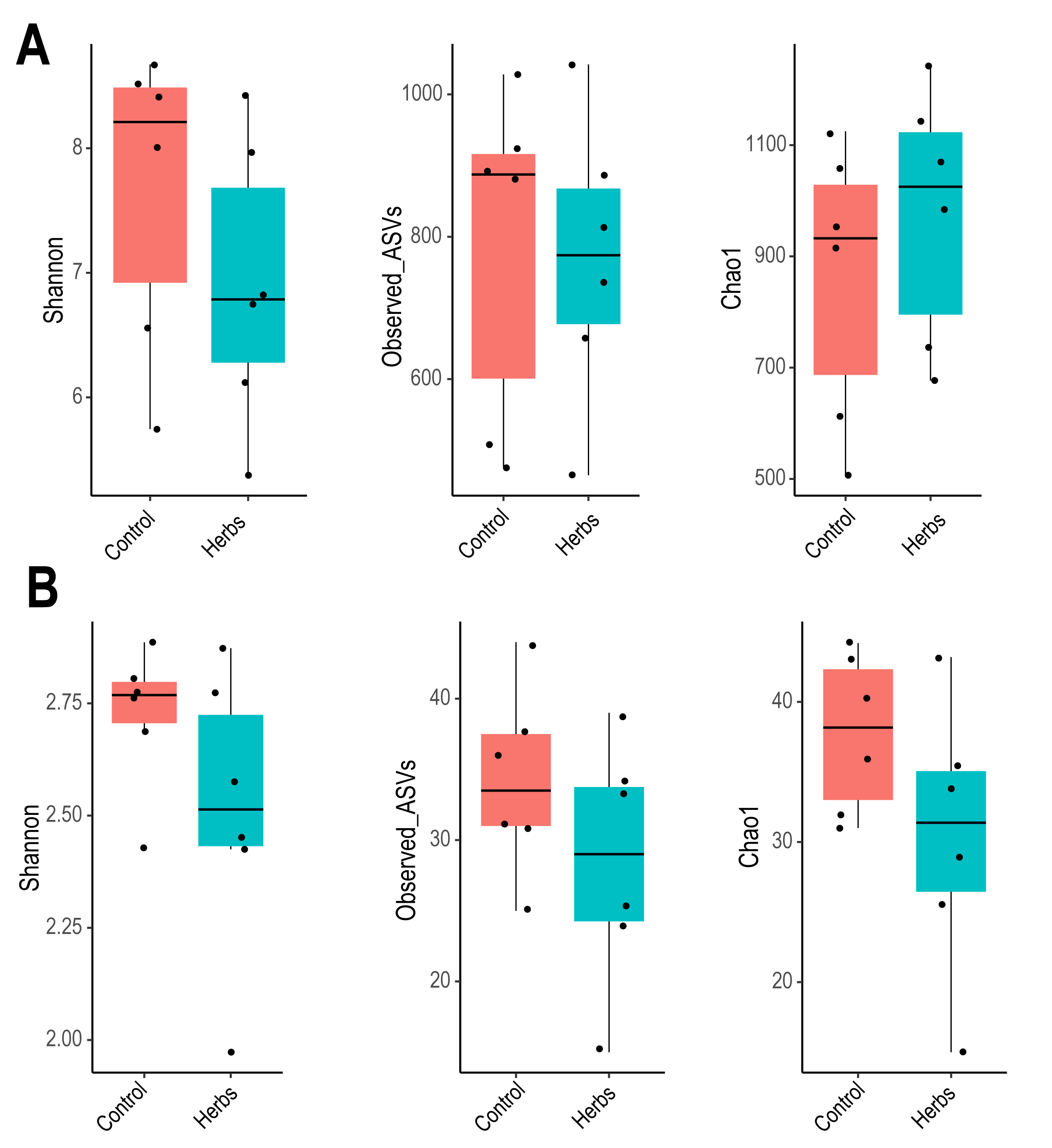

Supplement: SUPPLEMENTARY FIGURE S2 — Comparison of alpha diversity metrics between the Control and Herbs groups for bacterial (A) and archaeal (B) communities. Diversity indices include the Shannon index, observed ASVs, and Chao1 index, illustrating microbial diversity and richness. No statistically significant differences were detected between the two groups. [file Image_2.tif]
