# Supplementary material for: Herbal formula alleviates heat stress by improving physiological and biochemical attributes and modulating the rumen microbiome in dairy cows
Source: Front Vet Sci. 2025 Mar 7;12:1558856. doi: 10.3389/fvets.2025.1558856 (PMC11925914; doi:10.3389/fvets.2025.1558856)
Supplement: Supplementary file 3 [file Table_1.docx]

**Table S1.** Ingredients and Chemical composition of total mixed ration

| Ingredient | Content, % of DM | Chemical composition | Content, % of DM |
| --- | --- | --- | --- |
| Silage | 54.12 | NE_L_^2^(MJ/kg) | 6.24 |
| High-moisture corn | 11.87 | Crude protein | 16.18 |
| Alfalfa hay | 10.18 | Ca | 1.32 |
| Oat grass | 3.40 | P | 0.42 |
| Cotton seed | 3.40 | Neutral detergent fiber | 35.29 |
| Soybean meal | 8.42 | Acid detergent fiber | 22.26 |
| Molasses | 0.74 |  |  |
| Premix^1^ | 7.87 |  |  |
| Total | 100.00 |  |  |

^1^The premix provided the following per kg of the dietary diet：VA，500,000 IU/kg；VD，90,000 IU/kg； VE, 2,200 IU/kg；Ca,196 mg/kg；P，16.0 mg/kg；Chromium propionate，40 mg/kg；Mg，5200 mg/kg；Se， 30 mg/kg；Fe，800 mg/kg；Zn，2500 mg/kg；I，36mg/kg；Co，40mg/kg.

^2^ NE_L_: Net energy of lactation, a calculated value according to NRC (2001), while the other nutrient levels were measured value.

**Table S2.** Primer sequences for RT-qPCR

| Primer name | Primer sequence (5'to3') | Product size（bp） |
| --- | --- | --- |
| HSPA1A Fwd | GACAAGTGCCAGGAGGTGATT | 115 |
| HSPA1A Rev | AGTCTGCTGATGATGGGGTTA |  |
| HSPA8 Fwd | TCAGCGTCAGGCTACCAAAG | 245 |
| HSPA8 Rev | TGTCAAAGTCTTCTCCACCCA |  |
| HSPA1B Fwd | AAGCACAAGAAGGACATTGCACCC | 130 |
| HSPA1B Rev | AAGTGTAGAAATCCACGCCCTCCT |  |
| SOD1 Fwd | TGCAGGTCCTCACTTTAATCC | 245 |
| SOD1 Rev | CAGCGTTGCCAGTCTTTGT |  |
| CAT Fwd | GGTGGGGCTCCAAATTACTA | 218 |
| CAT Rev | TGTGCGTCTTTCAGATGGC |  |
| GPX1 Fwd | CATCGCTCTGAGGCACAAC | 218 |
| GPX1 Rev | ATTCACCTCGCACTTTTCG |  |
